# Dectin-1 in the control of Th2-type T cell responses

**DOI:** 10.14800/rci.1094

**Published:** 2016-01-04

**Authors:** Katherine Upchurch, SangKon Oh, HyeMee Joo

**Affiliations:** 1 Baylor Institute for Immunology Research, Dallas, TX 75204, USA; 2 Baylor University, Institute for Biomedical Studies, Waco, TX 76706, USA

## Abstract

Dendritic cells (DCs) are major antigen-presenting cells (APCs) that can induce and control host immune responses. DCs express pattern recognition receptors (PRRs), which can translate external and internal triggers into different types of T cell responses. The types of CD4^+^ T cell responses elicited by DCs (e.g., Th1, Th2, Th17, Th21, Th22 and regulatory T cells (Tregs)) are associated with either host immunity or inflammatory diseases, including allergic diseases and autoimmune diseases. In particular, the pathogenic functions of Th2-type T cells in allergic immune disorders have been well documented, although Th2-type T cell responses are crucial for immunity against certain parasite infections. Recent evidence also indicates that the inflammatory Th2 signatures in cancers, including breast and pancreatic cancers, are highly associated with poor clinical outcomes in patients. It is thus important to find cellular/molecular targets expressed in DCs that control such inflammatory Th2-type T cell responses. In a recent paper published in *The Journal of Immunology*, we demonstrated that Dectin-1 expressed on the two major human DC subsets, myeloid DCs (mDCs) and plasmacytoid DCs (pDCs), has opposing roles in the control of Th2-type CD4^+^ T cell responses. Dectin-1 expressed on mDCs decreases Th2-type CD4^+^ T cell responses, while Dectin-1 expressed on pDCs favors Th2-type CD4^+^ T cell responses. This finding expands our understanding of the roles of DCs and Dectin-1 expressed on DCs in the pathogenesis of Th2-associated diseases and in host immunity to microbial infections and cancers.

DCs are major antigen-presenting cells (APCs) that can efficiently induce and activate host immune responses. DCs are also able to control immune responses towards immunity or tolerance ^[1, 2]^, partly due to their ability to control the types of antigen-specific CD4^+^ T cell responses. Such functional plasticity of DCs for the control of the quality and magnitudes of T cell responses is largely dependent on activation signals delivered through a variety of pattern recognition receptors (PRRs), including TLRs, retinoic acid-inducible gene 1 (RIG-I)-like receptors, nucleotide-binding oligomerization domain receptors (NOD-like receptors) and C-type lectin receptors. Recent studies have shown that PRR-mediated activation of other cell types, particularly cells surrounding DCs in tissue environments, can also promote functional plasticity of DCs to control the type of CD4^+^ T cell responses.

Dectin-1, a C-type lectin receptor, is known to be expressed on myeloid DCs (mDCs) ^[3, 4]^ but not plasmacytoid DCs (pDCs) ^[5, 6]^ in humans. It delivers intracellular signals and activates DCs, resulting in the initiation of immune responses ^[3, 7–9]^. After recognition of β-glucans ^[10]^, the tyrosine residue within the cytoplasmic immunoreceptor tyrosine-based activation marker (ITAM) motif is phosphorylated ^[11, 12]^, followed by the recruitment of spleen tyrosine kinase (Syk) and caspase recruitment domain-containing protein 9 (CARD9) ^[13–20]^. Such sequential signaling cascades give rise to the activation of nuclear factor of activated T cells (NFAT) ^[21]^, mitogen-activated protein kinase (MAPK) ^[22]^ and nuclear factor kappa-light-chain-enhancer of activated B cells (NF-κB) ^[12, 13, 23, 24]^. The activation of Dectin-1 signaling pathways can thus lead to the release of cytokines ^[3]^, including IL-10, IL-6, TNFα, and IL-23 ^[14, 23]^. These cytokines can directly influence the quality of T cell responses and, in particular, Dectin-1-mediated DC activation promotes Th17 cell differentiation ^[3, 14, 25, 26]^. In accordance, human memory T cell responses to fungal antigens are mainly Th17- ^[13, 14]^, with a lesser involvement of Th1-type T cell responses ^[27, 28]^. Cytokines, such as IL-6 and IL-1β, released by Dectin-1-activated antigen-presenting cells (APCs) enhance the induction and activation of IL-17-producing T cell responses ^[13, 14, 19, 29]^, which are essential for host defense against certain fungal and bacterial infections ^[13, 14, 30, 31]^

Dectin-1 contains an internalization signal sequence for the lysosomal endosome ^[32, 33]^ and thus phagocytosis of β-glucan particles by Dectin-1 can contribute to antigen-specific T cell responses. Recently, Carter *et al*. ^[26]^ showed that ovalbumin (OVA)-transgenic mice immunized with OVA conjugated anti-Dectin-1 antibody induced potent CD4^+^ T cell responses, but weak CD8^+^ T cell responses. On the other hand, Leibundgut-Landmann *et al*. ^[34]^ showed that murine DCs stimulated with β-glucans were able to prime cytotoxic CD8^+^ T cell responses. These studies suggested that Dectin-1 mediated antigen delivery could result in both antigen-specific CD4^+^ and CD8^+^ T cell responses. Consistently, in our recent study, we showed that antigen targeting to human DCs via Dectin-1 results in antigen-specific CD4^+^ and CD8^+^ T cell responses ^[19, 35]^. In addition, we demonstrated that antigen targeting to DCs via human Dectin-1 (hDectin-1) along with TLR2 ligands could promote antigen-specific Th17 responses in humans ^[19]^. In line with these observations, β-glucans, as well as an agonistic anti-hDectin-1 antibody, can activate DCs to produce multiple cytokines, including IL-6, IL-1β, and IL-10 ^[9, 35, 36, 37, 38]^.

In humans, Dectin-1 is expressed not only on mDCs and macrophages, but also on B cells, neutrophils, and eosinophils ^[4]^, suggesting that Dectin-1 expression in humans is not myeloid restricted. Therefore, we re-investigated hDectin-1 expression and function on pDCs and compared them with those of mDCs ^[38]^. In contrast to previous reports ^[5, 6]^, we found that pDCs from the blood of healthy individuals express Dectin-1, although the surface expression levels of Dectin-1 on pDCs from blood, tonsils, and spleens were not similar. We also found that the expression levels of surface Dectin-1 on pDCs from the blood were highly variable among healthy subjects, suggesting that Dectin-1 expression on human pDCs could be controlled by factors that need to be further characterized. Dectin-1 expression in human pDCs was further confirmed at the RNA level, where we determined two major isoforms (A and B) and one minor isoform (D) of Dectin-1 in pDCs. We further reported that Dectin-1 expressed on pDCs could deliver activation signals via Syk, resulting in pDC surface maturation as well as cytokine (IL-6, TNFα, and IFNα) and chemokine (IP-10, MIP-1α, MIP-1β, and IL-8) expression. Unlike mDCs stimulated with house dust mite *Dermatophagoides farina* (Df) via Dectin-2, pDCs stimulated with β-glucan did not secrete cysteinyl leukotrienes (Cys-LT), which has been associated with Th2 immunity to inhaled allergens ^[39]^. Instead, pDCs activated via Dectin-1 expressed IFNα, which can induce the expression of OX40L that in turn promotes Th2-type T cell responses. This clearly illustrates that pDCs have unique functions to promote Th2 responses, particularly in the presence of Dectin-1 ligands.

OX40 ligand (OX40L) is known to be mainly expressed by APCs, although it is not constitutively expressed. It can also be induced in various other cell types, including endothelial cells and T cells ^[40, 41]^. The receptor for OX40L, OX40, is mainly found on activated CD4^+^ and CD8^+^ T cells. When expressed alongside a co-stimulatory molecule on APCs, OX40L is able to prolong T cell survival and increase T cell cytokine production ^[41]^. One of the most well defined modulations of OX40L expression on DCs is through thymic stromal lymphopoietin (TSLP). TSLP is a cytokine produced by epithelial cells, especially those in the lung, skin and gut, but also may be produced by fibroblasts, smooth muscle cells and mast cells ^[42, 43]^. TSLP-treated mDCs express high levels of CD86, a co-stimulatory marker, as well as OX40L. When these TSLP-DCs are co-cultured with CD4^+^ T cells, the T cells give rise to inflammatory Th2 cells, producing high levels of IL-4, IL-5, IL-13, and TNFα ^[43–45]^.

In contrast to the roles of Dectin-1 expressed on human pDCs, mDCs activated via Dectin-1 significantly decrease Th2-type CD4^+^ T cell responses ^[38]^. This applies to both induction and activation of naïve and memory CD4^+^ T cell responses. We further demonstrated that Dectin-1-activated mDCs secrete IL-10, which contributes to the suppression of OX40L expression. This is followed by decreased Th2-type T cell responses ^[38]^. Such contrasting roles of Dectin-1 expressed on the two major subsets of human DCs might have important implications in inflammatory Th2-associated allergic immune disorders as well as in certain types of cancers, although further studies need to be performed in the context of such diseases. Meanwhile, data from our study ^[38]^ suggest that Dectin-1 expressed on mDCs could be a novel target to suppress the induction as well as activation of such inflammatory Th2-type T cell responses.

## Abbreviations

APC: antigen-presenting cell
BDCA-2: blood dendritic cell antigen-2
CARD9: caspase recruitment domain-containing protein 9
Cys-LT: cysteinyl leukotriene
DC: dendritic cell
hDectin-1: human Dectin-1
IFN: interferon
IL: interleukin
ITAM: immunoreceptor tyrosine-based activation motif
MAPK: mitogen-activated protein kinase
MCP-1: monocyte chemoattractant protein-1
mDC: myeloid DC
MHC: major histocompatibility complex
NFAT: nuclear factor of activated T cells
NF-κB: nuclear factor kappa-light-chain-enhancer of activated B cells
NOD: nucleotide-binding oligomerization domain
OVA: ovalbumin
OX40L: OX40 ligand
PAMP: pathogen associated molecular pattern
pDC: plasmacytoid DC
PRR: pattern recognition receptor
RIG-1: retinoic-acid-inducible protein 1
RNA: ribonucleic acid
Syk: spleen tyrosine kinase
TLR: toll-like receptor
TNFα: tumor necrosis factor α
TSLP: thymic stromal lymphopoietin
